# Trauma-Based Sexually Dimorphic Changes in the Connectome and Its Association with Central Sensitization Syndromes—A Systematic Review

**DOI:** 10.3390/brainsci14111105

**Published:** 2024-10-30

**Authors:** Nicole Quodling, Shad Groves, Norman Hoffman, Frederick R. Carrick, Monèm Jemni

**Affiliations:** 1 Department of Neurology, Carrick Institute, Cape Canaveral, FL 32920, USA; shadgroves@gmail.com (S.G.); drnorm@hoffmanwellness.com (N.H.); drfrcarrick@post.harvard.edu (F.R.C.); monemj@hotmail.com (M.J.); 2 Centre for Mental Health Research in Association with the University of Cambridge, Cambridge CB2 1TN, UK; 3 Neurology, University of Central Florida College of Medicine, Orlando, FL 23816, USA; 4 Burnett School of Biomedical Science, University of Central Florida, Orlando, FL 32827, USA; 5 MGH Institute for Health Professions, Boston, MA 02129, USA; 6 Faculty of Physical Education, Ningbo University, Ningbo 315000, China

**Keywords:** chronic pain syndromes, fibromyalgia, neuropathic pain, prenatal and early life adversity

## Abstract

Background/Objectives: Chronic pain syndromes pose a significant global health challenge to patients and physicians with a complex relationship of biological and psychosocial factors that are only partly understood. Emerging research suggests an association between prenatal and childhood adversity and the development of somatic syndromes, particularly in females. This study aims to explore the relationship between sexual dimorphic epigenetic changes in the connectome and prenatal and early life adversity (ELA). Methods: A review of the existing literature was conducted, examining studies utilizing MRI to identify critical periods of environmental influence on neural phenotypes. Results: The findings indicate a significant association between prenatal and childhood adversity and the emergence of central sensitization syndromes, particularly among females. Notably, alterations in grey matter volume and neural connectivity patterns were observed, suggesting that early adverse experiences can influence pain signaling mechanisms. Conclusions: Understanding the role of sex differences in brain circuitry is crucial for developing personalized pain management strategies. This study highlights the importance of considering both biological and psychosocial factors in addressing chronic pain, as interventions based predominantly on male subjects may be less effective for females. Further research is warranted to explore these differences and refine therapeutic approaches.

## 1. Introduction

Chronic pain syndromes represent a complex interaction of biological and psychosocial factors that are only partly understood, posing a challenge to patients and physicians [1,2]. Chronic pain, defined as the perception of pain greater than three months, impacts quality of life, creating a significant global health issue with a prevalence in lower- and middle-income countries thought to be 20–60% of the population [2,3,4,5,6,7,8,9]. Sensitization of the central nervous system may contribute to the understanding of the pathogenesis and maintenance of chronic widespread pain [1,5,10]. The term central sensitization is a condition that is associated with neuroplastic changes in the central nervous system (CNS), whereby the perception of pain is maintained even when the initial nociceptive insult has resolved [11]. Central sensitization is associated with CNS changes, including chronic upregulation of nociceptive receptors. One established theory is that chronically increased nociception may lead to increased levels of substance P being released, lowering thresholds for perceived pain [5]. Substance P is a neurotransmitter released from primary afferent unmyelinated C-fibers related to the sensitization of nociceptive pathways [12]. Recent research has found that central changes affect neurotransmitter receptors, leading to decreased endocannabinoid sensitivity, for example. Such changes contribute to the hyperexcitation of pain processing pathways, leading to allodynia, hyperalgesia, and cognitive, affective, and behavioral changes [12,13]. Fibromyalgia is a prototypical chronic centralized pain condition that affects the entire musculoskeletal system and is characterized by hyperalgesia and allodynia without any apparent peripheral tissue damage, combined with fatigue, sleep, cognitive, and mood problems [1,14,15,16,17,18]. Complex regional pain syndrome (CRPS) is a chronic pain disorder characterized by spontaneous or regionally evoked pain and trophic changes typically affecting the distal extremities, particularly the upper limbs. Whilst CRPS usually develops after a peripheral event, it is likely maintained by changes in the central nervous system [11]. Alterations in grey matter volume and functional neural connectivity have been demonstrated in various conditions of central sensitization and medically unexplained pain [5,19]. Chronic somatic and visceral syndromes are often comorbid with each other and with internalizing symptoms of anxiety and depression [1,5,10]. The early-life risk markers for developing central sensitization syndromes include genetic factors, prenatal stress and birth trauma, early life adversity (ELA), female sex, injury, sleep disorders and lifestyle disorders [9,13,14,20,21,22,23,24].

ELA in childhood can come in the form of negative experiences, e.g., abuse or trauma, or the absence of experience, e.g., neglect or deprivation [25,26]. Adverse childhood experiences (ACEs) are repeated aversive experiences that represent deviations from the expected environment and require adaptation [27]. These associations include perinatal exposure to substance abuse, maternal deprivation, growing up with a depressed parent, psychological trauma, physical or verbal abuse from parents, and physical or sexual abuse by an adult [28]. Childhood maltreatment (CM) includes highly stressful and potentially traumatic events or situations that occur during childhood and/or adolescence, which include sexual abuse, psychological abuse, and neglect [29]. CM moderates the association between an adult traumatic event and adult psychopathology, such that those who experienced CM have more severe symptoms after later trauma than those who did not experience maltreatment [30]. Chronic pain has also been shown to be more prevalent in individuals exposed to ELA than in the general population [12,20]. The quality of parental care, nutrition, cognitive stimulation, and socioeconomic status during early child development have been shown to affect brain morphology and functionality throughout the life course [27,31]. Understanding embryological neurodevelopment may improve our understanding of the developmental origins of disease [32].

The fetal maternal environment and that of early childhood are essential modulators of brain development, with consequences throughout childhood and the lifespan [20,33,34]. Brain regions undergoing extensive neurogenesis are particularly vulnerable to insults because developmental patterns are being established [35]. Glucocorticoid exposure might induce these long-term changes by acting as epigenetic modulators interfering with transcription factors. DNA methylation embeds the impact of early life experience in the genome so that environmental perturbations can modify the phenotype of the offspring [33]. Advances in neuroimaging have expanded the concept that the nervous system is a structurally interconnected and integrated network of neuronal pools, allowing the influence of one neural system over another [36]. Adults with chronic pain who have experienced ACE exposure can show different brain alterations than adults with chronic pain who have not experienced ACE [20]. These areas include the prefrontal cortex (PFC), superior temporal gyrus, insula, amygdala, hippocampus, putamen, and the anterior cerebellum [27,31]. Glucocorticoids released in response to stress bind to glucocorticoid and mineralocorticoid receptors, causing changes in DNA methylation, which correlate with an enhanced responsivity to a second stressful challenge [33]. Thus, prenatal and early life stress can render offspring more susceptible to additional environmental exposures later in life, resulting in the unmasking of psychopathology [20,30,35]. However, the maternal environment and genetic profile are not the only contribution to embryonic development. The paternal genome is demethylated faster in the first days of embryo development than the maternal genome, so preconceptual paternal stress may also significantly impact the embryo [25].

There has been an association between prenatal and childhood adversity and the development of somatic syndromes [5,12,20,35,37], predominantly in females [9,17,19,38,39,40,41,42,43,44,45]. However, the reasons for female predominance are mainly unknown [28]. Fetal sex may mediate stress responsiveness [12,19,32,46]. Prenatal trauma and ELA have been shown to put male offspring at risk of developing socialization and externalizing disorders [47]. In contrast, female offspring exposed to ELA appear to be at risk of internalizing disorders [34,47,48]. This review describes the effects of prenatal and early childhood adversity on brain development. One focus is on studies characterizing human brain development using MRI to identify sensitive periods during which the environment influences the adult phenotype. A further area of interest is to identify biological mechanisms contributing to vulnerability and resilience to stress, especially concerning sex assigned at birth.

Therefore, the main objective of this review is to investigate the association between sexually dimorphic changes in the connectome induced by prenatal difficulty and ELA and the development of central sensitization syndromes.

## 2. Methods

**Figure 1:**
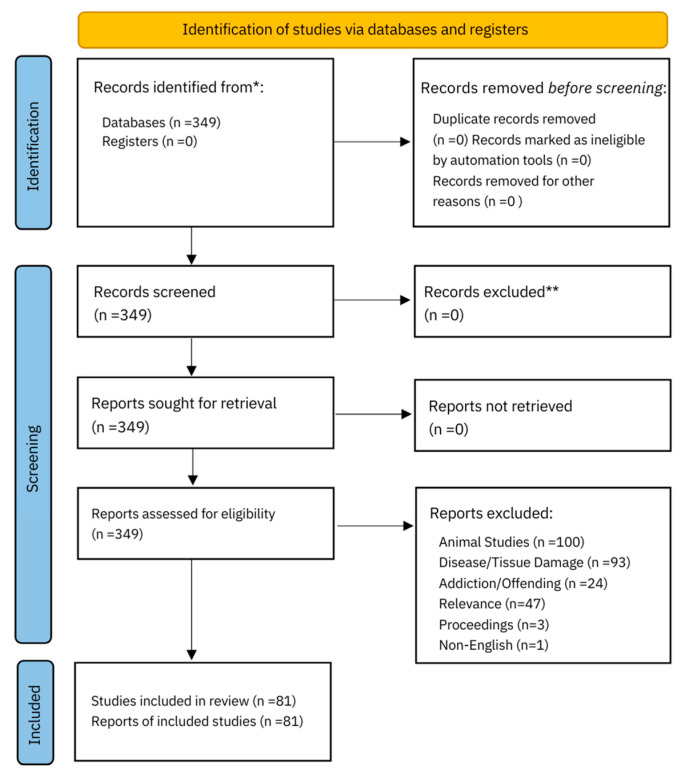
PRISMA 2020 flow diagram for new systematic reviews which included searches of databases and registers only [49] for more information, visit http://www.prisma-statement.org/. * Consider, if feasible to do so, reporting the number of records identified from each database or register searched (rather than the total number across all databases/registers).** If automation tools were used, indicate how many records were excluded by a human and how many were excluded by automation tools.

The literature review was conducted according to the PRISMA framework (Figure 1), The search was performed using the PubMed database and the keywords: central sensitization, fibromyalgia, complex regional pain syndrome, and neuropathic pain combined with the keywords prenatal trauma, early life adversity, and childhood maltreatment. The terms connectome and sexual dimorphism were then applied to all previous keywords. CRPS and fibromyalgia were included in the search terms as they are among the most common somatic medically unexplained pain syndromes encountered in practice. PubMed was chosen because it is accessible, user friendly, and uses synonymous search terms. Articles within the last ten years, from 2013 to 2023, were included. Papers were excluded if they were animal studies, investigated tissue damage, disease processes or addiction, were conference proceedings, or non-English. One paper was excluded because it used EEG procedures. Papers elicited from the search on sexual dimorphism were marked irrelevant if there was no reference to sex assigned at birth comparison in the text. Previous reviews were included to summarize evidence from different outcomes, conditions, or populations.

## 3. Results and Discussion

In total, 349 studies on conditions of central sensitization, the connectome, and sexual dimorphism were identified. After title, abstract, and full-text screening, 81 studies were identified as meeting the inclusion criteria.

### 3.1. Association of Adverse Childhood Experiences with Central Sensitisation

Published systematic reviews have demonstrated positive associations between ELA and the subsequent development of somatic and visceral syndromes, with the risk of developing somatic syndromes being higher [5]. There is a high prevalence of posttraumatic stress disorder (PTSD) (37.3%) among fibromyalgia patients, significantly higher than that observed among other chronic pain patients, for example, rheumatoid arthritis [31,39].

Chronic pain is characterized by the disruption of whole-brain functional connectivity globally and the disruption of local connectivity [26,28,50,51]. The pain experience is highly subjective and top-down modulated [43]. The default mode network (DMN) is the primary network related to chronic pain and comprises the posterior cingulate cortex (PCC), medial prefrontal cortex (mPFC), and lateral parietal lobe [45,52,53]. The reward-motivation network (including the PFC, nucleus accumbens, hippocampus, and ventral tegmentum) and the descending pain modulatory system (anterior cingulate cortex (ACC), amygdala, and hypothalamus) are also implicated in vulnerability to painful conditions along with the insula and thalamus, which are involved with pain perception [27,52,54]. In addition to encoding pain intensity and duration, the structure of the neuraxis plays an integral part in developing chronic pain. The most consistent earlier results regarding perturbations of the resting state point to changes in functional connectivity between the insula and the DMN [20,38] and between the insula and the mPFC [51,55]. Maltreated children show significant reductions in both global connectivity and local connectivity with reported reduced cortical thickness within regions involved in emotional regulation, including the orbitofrontal cortex (OFC), ACC and mPFC [5,20,45,46,50]. Threat and deprivation may show opposing effects where deprivation is associated with reductions in association and prefrontal cortical thickness, and threat affects the connectivity of areas involved in emotional learning, including the hippocampus, amygdala, and PFC [55]. Timing, chronicity, and maltreatment type are critical factors that likely shape neural development and behavioral outcomes [23,50], such that children who endured more extended periods of hardship and higher levels of verbal and domestic abuse present with more significant alteration [45,46]. Whether alterations in brain function precede or result from chronic pain syndromes, they might lead to a cycle of decreased resilience [28]. Connectivity is also altered with current pain states [56], with treatment induced changes demonstrated with pain reduction [57].

### 3.2. Association of Adverse Childhood Experiences with Connectome

The quality of parental care, nutrition, cognitive stimulation, and socioeconomic status during early child development have been shown to affect brain morphology and functionality throughout the life course [12,23,25,26,31,46,50,55,58,59,60]. Graph-based network analysis is utilized to reveal information about the topography of human brain networks by characterizing different brain regions as nodes and white matter tracts reconstructed through probabilistic fiber tracking as connections between the nodes as edges [46,52]. This framework allows the degree of functional segregation and integration of the network to be investigated and yields invaluable insights into normative brain development [46]. Deviations from small world brain architecture, considered the most efficient network organization due to its dense local clusters of nodes connected by short paths facilitating quick information processing, indicate several neurodevelopmental, psychiatric and neurological disorders [46]. Studies have employed a unitary measure that assesses the whole-brain degree rank order disruption (kD), defined as the gradient fitted to the mean difference in nodal degree between any given subject or group of subjects about the mean nodal degree in a control population using normalized mutual information (NMI). The overall similarity of subjects to the off-site control community is determined using post-hoc analysis [26]. Changes in kD have been shown to be proportional to reported pain intensity but only once pain became chronic, after approximately one year of persistent pain [51].

Adults with chronic pain who have experienced ACE exposure can show different brain alterations than adults with chronic pain who have not experienced ACE [27]. These areas include the superior PFC (sPFC), superior and inferior temporal cortex, insula, left lingual gyrus, hippocampus, putamen, and anterior cerebellum [31,61].

### 3.3. Parts of the Connectome Implicated in Prenatal and Childhood Trauma

#### 3.3.1. The Cerebrum

Alterations in PFC grey matter have been reported in multiple chronic pain conditions [6,54], although the direction of this association is unknown [61]. The PFC influences the descending regulation of neuronal activity of the dorsal horn of the spinal cord, thereby influencing nociception [57]. The mPFC provides the primary source of cortical input to the periaqueductal grey (PAG) and is thought to play an integral role in descending pain modulation [32,62]. Heightened mPFC activity when processing reward or social exclusion in individuals with higher levels of ELA is associated with learned helplessness and other depression-like behavioral deficits after exposure to stress [63], with increased grey matter volumes of the mPFC observed in maltreated individuals [29]. Reward sensitivity and anticipation are negatively impacted in ELA [20,50] and are mediated by the OFC, influencing the perception of pain [26,27,46,57].

The dorsolateral PFC is known to perform various cognitive functions, including working memory, motivation, and attentional control [60] and the processing of pain [56,64]. Cerebral blood flow and cortical connectivity are increased within the dlPFC of patients with chronic pain [12,54], with decreased grey matter volume of the dlPFC associated with neighborhood poverty [59]. Cortical thinning in the dlPFC provides further evidence for the involvement of the dlPFC in top-down pain modulatory processes via its connections to other pain modulatory brain areas including the PAG [57]. Other areas that might not affect the risk of developing chronic pain but are relevant to pain perception include changes in the primary and secondary somatosensory cortex, a significant area regarding the sensation and perception of pain and episodic memory retrieval. Individuals with major depressive disorder and those who have experienced early life adversity have been found to have abnormal activity in this region [27] and a significant increase in regional cerebral blood flow has been observed in the primary somatosensory cortex of migraineurs compared with healthy controls [13]. The parietal lobe is a significant area in processing pain because of its role in processing sensation [27]. The interaction between stress and ELA is related to alterations during emotional processing, mainly in the middle temporal [26] and supramarginal gyri [58]. Decreased grey matter volume has been observed in the right middle temporal and left lingual gyri associated with cognitive and affective disorders [6,29].

The insula is activated by ELA and is an essential area concerning pain perception. There is increased insula activation in children exposed to violence [26,27] and increased connectivity with the insula and DMN in chronic pain [20,38]. Interoceptive awareness is mediated by the right anterior insula connectivity to structures including the amygdala, hippocampus, OFC and precuneus [36]. Pain responses in the insula seem enhanced in men with chronic pain, with the insula volume negatively correlated with perceived personal control of the condition [61].

#### 3.3.2. The Limbic System

Exogenous glucocorticoids reduce ACC activation and simultaneously increase negative affect [27,46]. Individuals with a history of severe maltreatment show a lower volume in the left ACC [26,55,58,60,65]. These changes may not increase the risk of developing chronic pain but could affect how pain is perceived [27,64], especially in women [61]. The subgenual anterior cingulate cortex (sgACC) is connected to the PAG, rostroventral medulla, and mPFC, critical components in the descending pain modulation pathway associated with reduced temporal summation of pain and improved habituation [32]. An fMRI study of static resting state functional connectivity has shown that females have greater connectivity of the sgACC with the PAG and raphe nuclei, while men have greater connectivity with the salience network [32]. Altered white matter properties in the PCC have been observed in abdominal pain conditions, including irritable bowel syndrome (IBS) and primary dysmenorrhea [38,53]. The hippocampus contributes to stress regulation via the hypothalamic-pituitary-adrenal (HPA) axis by providing inhibitory feedback [59]. Estrogen may enhance choline uptake, acetylcholine synthesis and blunt cortisol responsivity, whereas progesterone may increase HPA axis reactivity to stress, altering autonomic and neuroendocrine homeostasis [66]. Glucocorticoids are toxic to the hippocampus, which might explain the hippocampal atrophy often observed in individuals with ELA [29,35,60,67] and chronic pain [6]. Smaller hippocampus volume may increase the vulnerability to anxiety disorders and posttraumatic stress [57]. The hippocampus is recruited in anticipation of pain and is associated with internalizing symptoms following maltreatment [19].

The amygdala affects affective processing and perceived pain [20,27,46]. ELA experiences have been associated with altered amygdala connectivity, including lower connectivity to the hippocampus, insula, OFC and postcentral gyrus and greater connectivity to the PFC [19,50]. Volume decreases in the amygdala have also been shown in studies of adults reporting ELA [60], with increased amygdala connectivity with the hippocampus and PFC during emotion processing [45]. Increased amygdala activity has been observed in females suffering from IBS [38] and chronic pain [61]. Visceral neural circuits converge on the paraventricular nucleus of the hypothalamus (PVN), bed nucleus of the stria terminalis (BST), and the amygdala to control autonomic and neuroendocrine stress responses through viscerosensory afferents to the nucleus tractus solitarius (NST). The NST also receives viscerosensory information from the vagus and relays it to the ventral BST and PVN. Research indicates a link between childhood adversity and visceral, stress-related circuits, contributing significantly to differences in stress reactivity, affective processes and response to threat [20]. The thalamus is a vital region in the integration of brain function and is believed to play a crucial role in the experience and expression of emotion and stress responses, influencing the perception of pain [27,57,60]. Abnormal corticothalamic connectivity suggests altered pain processing in migraines [68]. Functional abnormalities in the thalamus are consistently reported in CRPS [11,54].

The caudate and the putamen are thought to play a role in pain and analgesia [27,57]. The caudate is involved in reward-related and emotional processing [29]. Volume decreases in the caudate [60] and increased white matter densities in the right putamen and globus pallidus have been demonstrated in ELA [27]

### 3.4. Mechanisms of Connectome Alteration

ELA, including in utero, can modify the epigenome, potentially leading to changes in DNA methylation and HPA axis activity [33,69]. Dysregulation of the HPA axis may affect nociceptive processing, predisposing individuals to sensitization and increased inflammation markers [5]. Neuroinflammation is implicated in neurodegenerative diseases, mood disorders and chronic pain [62,70,71,72]. Chronic cortisol secretion can affect dendritic and axonal sprouting in glucocorticoid-receptor-rich areas, diminishing white matter integrity [73]. The HPA axis has also been implicated in the observation that ELA accelerates telomere length shortening [74], associated with cellular damage [75]. A linear relationship has been demonstrated between parental care and telomere length, suggesting that higher parental care may protect against telomere shortening in the presence of subsequent stressors [74].

Deprivation may shape neural development via activity-dependent plasticity, leading to increased apoptosis of synaptic connections, particularly within association cortices [20]. ELA may also activate stress reactivity and nociception through hyperalgesic priming. Thereby, pain is a stressor that may generate a feedback loop impacting stress regulation [28].

Sexual dimorphism may be due to different gene expressions because of variability in the hormonal environment of the two sexes [7]. The transient receptor potential (TRP) channel family controls cellular differentiation by regulating gene expression through activating calcium-dependent transcription factors. The DNA methylation of TRP channels is implicated in the pathology of pain syndromes. Steroids and TRP channels also closely interplay with estradiol positively and androgens negatively, enhancing TRP expression. Therefore, TRP channels may function as a modulator of sexual dimorphism in pain perception [4,33].

### 3.5. Sexual Dimorphism

Prenatal difficulty and ELA may influence the epigenome in a sex-dependent manner, which might underpin the higher rates of chronic pain in females [19,55]. ELA has been associated with sexually dimorphic altered connectivity within the brain’s fear-regulatory circuit, including the hippocampus, amygdala, and sgACC, as a component of the ventromedial PFC (vmPFC). The vmPFC inhibits hyperactivity of the amygdala with a consequent expression of fear responses, whereas the hippocampus inhibits fear responses via connections to both the amygdala and vmPFC [12,19].

### 3.6. Sex Assigned at Birth and the Limbic System

There are more significant growth rates for both the amygdala and hippocampus in females during the first several years of life, with a more extended period of growth in the amygdala for males [19]. As periods of rapid brain maturation are susceptible to ELA, there may be a more significant neural impact of maltreatment in females by affecting the connectivity of the amygdala and hippocampus with the sgACC [35]. This alteration in amygdala connectivity to the cingulate cortex may explain higher internalizing symptoms in females [25]. Sex differences in sgACC functional connectivity have also been observed in chronic pain patients [32].

However, exposure to trauma has a relatively more significant effect on hippocampal volume in males than in females, as estrogen exhibits neuroprotective effects in the hippocampus [19]. Males show greater structural connectivity between the vmPFC and hippocampus, which may offer protection against CM. However, adult trauma has revealed increased hippocampus–vmPFC connectivity, with failure to increase this connectivity associated with PTSD. This observation emphasizes the significance timing of trauma has on the connectome, with age-dimorphic effects also revealed in the literature [32,34].

The interaction of the HPA, sympathetic nervous system and immune system can also contribute to the effects of ELA. Women show greater activation of the HPA [5,30,35,66], higher sympathetic tone and greater levels of inflammation [32,35,76,77,78]. Table 1 summarizes examples of sexually dimorphic experiences of central sensitization syndromes.

### 3.7. Sex and Adverse Childhood Experiences

Sexually dimorphic effects of trauma may also be unrelated to biological differences in neural development. Females and males may be exposed to different types or higher rates of stressors, which may correspond to the sex differences noted in patients with chronic pain and comorbid depression [12,25,47,79,80]. Dysmenorrhea may also predispose women to a chronic pain state. Altered white matter integrity of the cingulum associated with dysmenorrheic pain may lead to spontaneous communication within the pain connectome, amplified by nociceptive input, so pain becomes a learned behavior [39]. Examples of the sexually dimorphic experiences of adverse childhood experiences revealed in the literature are summarized in Table 2.

## 4. Conclusions

The main objective of this review was to investigate the association between sexual dimorphic epigenetic changes in the connectome induced by prenatal difficulty and ELA. A secondary objective was to examine any potential association of ELA with the development of central sensitization syndromes. The results showed that brain regions undergoing extensive neurogenesis are particularly vulnerable to insults. However, the type of adversity, e.g., deprivation and threat, and the age at which it was experienced impact the connectome differently and potentially affect the sexes differently. Alterations in the connectome associated with trauma can include a grey matter volume decrease, higher or lower integrity of white matter tracts, and higher or lower gyrification indices. The brain is programmed for survivability, so increasing tracts associated with, for example, hypervigilance, can be seen as advantageous whilst placing the individual at risk for anxiety disorders. Nevertheless, this is still a new field of study. Extensive literature reviews are invaluable for the comparison, collaboration, and assessment of information. Characterizing the effects of sex on brain circuitry is essential in developing effective personalized pain treatments because therapies designed with research based primarily on one sex can potentially be less effective in the other sex.

## 5. Limitations

Despite the structured search strategy, this review has limitations. An inherent recall and response bias is associated with self-reported documentation of childhood trauma, which may be unstable over time [30]. Not all gender identifications and sexual orientations are acknowledged or assessed. Despite the strengths of graph theory, another limitation of concern to developmental neuroscientists is the reliability of connectivity measures obtained from MRI scans interpreting graph metrics, which have shown limited test–retest reliability [55]. The origin of the information in the attached tables has not been well defined. The search was limited to ten years because of resource constraints.

## 6. Further Research

The influence of prenatal and childhood adversity on the developing connectome and its association with the adult phenotype is an emerging field, and further research will be required to prove or disprove these theories.

## Figures and Tables

**Table 1:** Sexually dimorphic experiences of central sensitization syndromes: facts and neuro-physiological mechanisms.

Authors	Facts
**Arout et al., 2018 [76]**	77,087 of 2,216,621 veterans with pain diagnoses were diagnosed with fibromyalgia and were three times more likely to be female (25.5% compared to 7.7%) and more likely to have multiple psychiatric comorbidities. In addition, females diagnosed with fibromyalgia were likely to be younger and more likely to have headaches, connective tissue diseases, and psychiatric comorbidities. In contrast, older males had more comorbid medical conditions. This study showed that there is a gender discrepancy between the types of chronic pain associated with females and males, with females having a higher probability of chronic pain secondary to conditions such as fibromyalgia.
**Camilleri, 2020 [38]**	A review examining differences between sexes in IBS, the brain-gut axis and sex hormones, epidemiology, pain perception, colonic transit, abdominal distension, overlapped with urogynecological conditions, psychological issues, anorexia, fibromyalgia, serotonin, and responsiveness to treatment of IBS. The authors found that in Western countries, the female to male ratio of IBS is 2:1, with a prevalence of 14.5% of females and 7.7% of males in the U.S. Householder Survey.
**Conversano et al., 2021 [39]**	A systemic review found that females with fibromyalgia tend to seek out medical care earlier than males with fibromyalgia.
**Henao-Pérez et al., 2022 [1]**	1,106 cases of fibromyalgia were reviewed with 295 females and 811 males. 42.6% of males with fibromyalgia suffered from depression and anxiety. The results also revealed a relationship between sex (female PR = 0.5 [0.28–0.86]) and low socioeconomic strata (PR = 0.53 [0.33–0.70]). This study concluded that there was a significant relationship between sex, whereby females were less likely to experience depression or anxiety than males with fibromyalgia but more likely to have low socioeconomic status.
**Jiang et al., 2020 [15]**	A questionnaire study with 668 patients with fibromyalgia (606 females) found a significant correlation between being of female sex and having a greater trigger point count. However, there were no sex-related differences in demographic characteristics, depression, anxiety, sleep problems, fibromyalgia symptom severity, cognitive dysfunction, and quality of life.
**Melikoglu & Celik, 2017 [8]**	80% of 70 individuals suffering from neuropathic pain presented poor quality of sleep (significantly higher scores of sleep latency, sleep duration, sleep efficiency, and daytime dysfunction). The Pittsburg Sleep Quality Index total was observed in patients with neuropathic pain compared to controls with factors including female sex and pain intensity. These findings suggest that females suffering from neuropathic pain will have a higher probability of impaired sleep.
**Nakua et al., [41]**	A sampling study stratified by population and age with a structured questionnaire found that the prevalence of both chronic back pain and chronic joint pain from arthritis was significantly higher in females compared to males. Females with primary education had a prevalence of chronic back pain of 36.2% (95% CI; 29.2, 43.3) and chronic arthritis/joint pain a prevalence of 15.8% (95% CI; 11.1, 20.6), while males had prevalence rates of 29.0% (95% CI; 23.4, 34.5) and 9.8% (95% CI; 6.4, 13.2) respectively. These findings support the hypothesis that there is the existence of sex differences between females and males in the prevalence of chronic pain.
**Petersen et al., 2020 [42]**	A stratified sample of 1590 participants including 943 females and 647 males found that the prevalence of having at least one functional somatic syndrome, including irritable bowel syndrome, fibromyalgia/chronic widespread pain, chronic fatigue syndrome, whiplash associated disorders, and multiple chemical sensitivity, was 9.3% (95% CI: 8.1–10.6), with all functional somatic syndromes more prevalent in females.
**Ruschak et al., 2023 [17]**	A scoping review concluded that the subjective perception and widespread pain are higher in females compared to males but that males would typically have more significant pathology, more painful experiences, and more catastrophic thoughts regarding their pain. Their findings support the hypothesis that there is a substantial difference in pain among the sexes and that females and males differ in their responses to pain, with both a greater sensitivity to pain and a higher risk of clinical pain more often observed among females but increased pain catastrophising in males.
**Söreskog et al., 2023 [2]**	A large retrospective observational cohort study with 480 males and 602 females treated with spinal cord stimulation found that the number of disability days varied considerably depending on age, sex, socioeconomic variables, and comorbidities. Male sex was associated with fewer net disability days.
**Toriyama et al., 2017 [13]**	A study of 176 episodic migraineurs compared to 132 age- and sex-matched controls found that risk factors associated with interictal widespread pressure hyperalgesia were female gender, younger age at migraine onset, higher frequency of migraine attacks, severe headache impact, cutaneous allodynia, and depression.
**Vagaska et al., 2019 [9]**	MRIs of the lumbar spine in 21 females and 21 males with chronic non-specific lower back pain were examined to evaluate for correlation with pain intensity and a predictor of neuropathic pain. While the study found no correlation between the severity of degenerative changes and pain, there were two independent predictors of neuropathic pain, including being female with an odds ratio of 11.9 and having a pain intensity of ≥4.5 in the previous 4 weeks with an odds ratio of 13.1. These findings support that females with chronic pain have a higher susceptibility to developing neuropathic pain regardless of tissue injury.
**Weimer et al., 2013 [44]**	17,583 veteran patients (1,945 female and 15,638 male) with moderate to severe chronic non-cancer pain were studied retrospectively. Females were more often diagnosed with two or more chronic pain conditions, including fibromyalgia, low back pain, inflammatory bowel disease, migraine headache, neck or joint pain, and arthritis (67% females vs 56% males). This study concludes that there is significant evidence that there is a sex difference present in chronic pain in female veterans compared to male veterans, regardless of diagnostic factors.
	Mechanism
**Larson et al., 2014 [62]**	A systematic review showed that females are less likely than males to recruit brown adipose tissue adaptations in response to chronic stress, correlating with a reduced body temperature, lower metabolic rates, and reduced circulating cortisol and corticosterone in response to stress; all hallmarks of fibromyalgia.
**de Kruijf et al., 2016 [6]**	Brain volumes measured in 3892 subjects found decreased total GMV in females with chronic pain, specifically in the temporal lobe, frontal lobe, and hippocampus in females, with no statistical differences observed in males.
**Neville et al., 2018 [16]**	In a study of 129 patients (68 females and 61 males) with osteoarthritis of the knee, 3.8% met the criteria of fibromyalgia, whereby females and males differed significantly in nearly every outcome, including fibromyalgia severity, clinical pain, anxiety, depression, and pressure pain sensitivity. In females, fibromyalgia scores significantly correlated with pressure pain sensitivity but not conditioned pain modulation or temporal summation, such that increased sensitivity was associated with greater fibromyalgia severity at all body sites examined. Additionally, as fibromyalgia scores increased, the association between pain sensitivity at the surgical knee and that at remote body sites also increased. No relationship between fibromyalgia score and quantitative sensory testing was observed in males. However males were shown to endure pain longer and have higher rates of anxiety and depression.
**Malfliet et al. 2019 [61]**	An MRI based study correlated brain grey matter morphology with self-reported psychosocial characteristics in 32 males and 62 females suffering from chronic spinal pain with perceived consequences, emotional representations, chronicity, and pain catastrophising. Males showed more significant associations of the precuneus cortex, precentral gyrus, and insula with perceived personal control and kinesiophobia. This study supports the findings that different grey matter morphological changes relate differently to psychosocial characteristics in females than in males.
**Doménech-García et al., 2022 [70]**	A RCT-based study found that there was a greater sympathetic vasomotor response contributing to expanding pressure-induced referral pain in females at multiple locations in the upper extremities, including the shoulder, arm, and forearm. These findings support the hypothesis that females are more susceptible to contributors of central sensitisation than males.
**Mejía-Terrazas et al., 2022 [7]**	Mutations in the voltage-dependent calcium channel gamma-2 subunit gene (CACNG2) were associated with neuronal hyperexcitability, including neuropathic pain. The authors concluded that specific alleles and genotypes could constitute severity markers in chronic peripheral neuropathic pain with a sex-biased effect.

**Table 2:** Neuro-physiological concepts underpinning the sexually dimorphic experience of adverse childhood experiences.

Author	Observations/Findings
**Bath, 2020 [25]**	This review highlights the importance of studying sex as a biological variable and understanding sex differences in response to ELA. It examines the historical and unfounded exclusion of female subjects from studies, chromosomal and hormonal effects on gene expression that contribute to sex selective effects on neurodevelopment and sex disparities in early postnatal care, where females may receive more significant levels of abuse. In contrast, males receive higher levels of maternal contact.
**Davis & Pfaff, 2014 [48]**	A review of 12 papers illustrating the neurodevelopmental consequences of foetal exposure to stress and stress hormones for males and females concluded that males have higher mortality, increased ASD rates, and females exhibit increased affective disorders, often unmasked during hormonal events.
**Enokido et al., 2014 [74]**	581 unrelated Japanese healthy subjects. Perceived parental care was assessed together with the leukocyte relative telomere length to determine the ratio of telomere/single copy gene. A multiple regression analyses showed shorter telomere length in males was related to lower scores of paternal care, while that in females was related to lower scores of maternal care.
**Erlich et al., 2021 [76]**	Examined links between child maltreatment and low-grade inflammation in adulthood in a sample of 155 low-income children (ages 8-12), half of whom had been exposed to maltreatment. Blood samples from children assessed C-reactive protein and cytokines, which were used to form a composite of low-grade inflammation. Analyses suggested that maltreatment exposure was associated with higher inflammation for females but not males. Females with exposures before the age of five had the highest low-grade inflammation and females who were exposed to two or more forms of maltreatment had higher inflammation compared to females who were not maltreated and had higher inflammation compared to girls who experienced one form of maltreatment. Males’ inflammation scores did not significantly differ as a function of the number of types of maltreatment they experienced.
**Gallo et al., 2017 [47]**	Participants in a population-based, birth cohort study in Pelotas, Brazil (N=3715) self-reported exposure to maltreatment (emotional abuse, physical neglect, physical abuse, sexual abuse, domestic violence) in confidential questionnaires at age 15 years, were assessed for major depression in interviews at age 18 years. Females exposed to emotional abuse and domestic violence were at increased risk for depression after adjustment for confounders and other types of maltreatment. Females exposed to two or more forms of maltreatment were at particularly high risk for depression compared with females not exposed to maltreatment. In adjusted analyses, maltreatment was not associated with depression for males. However, for both sexes, exposure to multiple forms of maltreatment (two or more types of maltreatment) increased risk of major depression.
**Ganguly & Brenhouse, 2015 [35]**	A review examines how early life adversity (ELA) has been associated with various psychopathologies and how sex differences contribute. A history of ELA was shown to increase the risk of developing a psychiatric disorder in adulthood. Female adolescents exposed to ELA expressed higher levels of IL-6 that forecasted higher cortisol and depression 6 months later. In females but not in males, increased HPA activity during childhood was shown to predict lower functional connectivity between the amygdala and PFC and the amygdala and hippocampus. Microglial colonization of the brain occurs much earlier in males than in females in the parietal cortex, hippocampus, and amygdala, which may contribute to distinct windows of neuroimmune vulnerability between males and females. Therefore, the sexes might be impacted by ELA in a gender specific manner, with females more vulnerable to early neuroendocrine-induced changes in corticolimbic circuitry and males more vulnerable to later neuroinflammation, possibly through microglial sensitisation.
**Martinez-Torteya et al., 2015 [69]**	Changes in infant cortisol levels from 7 to 16 months of age and the effects of known correlates of HPA axis activity, including sex, temperament, maternal psychopathology, maternal parenting, and demographic factors were examined. The authors used validated, age-appropriate stress induction tasks with a sample of infants whose mothers were (74%) or were not (26%) victims of maltreatment during their childhood. Participants were 167 mother–infant dyads (56% male infants) drawn from a larger longitudinal study of stress during childbearing years (*n* = 269). Infants did not show a cortisol response to psychosocial stress at 7 months but displayed reactivity at 16 months. Infant sex at 7 months significantly moderated the changes in cortisol secretion patterns. Females baseline cortisol levels declined more than males from 7 to 16 months, possibly reflecting differential maturation of the HPA axis and is consistent with previous findings of enhanced behavioral regulation in toddler females relative to males.
**McQuaid et al., 2019 [72] **	Examined the moderating role of three independent cytokine single nucleotide polymorphisms (SNPs; IL-1β rs16944, IL-6 rs1800795 SNP, TNF-α rs1800629) in the relationship between ELA and depressive symptoms, and whether these relationships were influenced by sex. The original sample comprised 925 Carleton University first year students, 343 females and 132 males (age range 17–35 yrs). The relation between childhood adversity and depressive symptoms was moderated by the IL-1β SNP. Among females, ELA was accompanied by elevated depressive symptoms irrespective of the IL-1β SNP, but among males, this relationship was pronounced for those carrying the GG genotype of the IL-1β SNP. Genetic variations of IL-1β functioning are related to depressive symptomatology associated with ELA and this may vary among males and females.
**Merrick et al., 2018 [80]**	Data were collected through the Behavioral Risk Factor Surveillance System. Of the 214 157 respondents included in the sample (51.51% female), 61.55% had at least 1 and 24.64% reported 3 or more Adverse Childhood Experiences (ACEs). Compared with male respondents, female respondents reported a greater prevalence of child sexual abuse (16.33% vs 6.70%), household substance abuse (28.72% vs 26.33%), and household mental illness (19.19% vs 13.71%).
**Morton & Ferraro, 2020 [77]**	This longitudinal study investigates whether childhood exposures influence adult chronic inflammation and mortality risk via adult health characteristics and socioeconomic status and whether gender moderates these relationships. A sample of 9,310 males and females over age 50 was analyzed and found that childhood socioeconomic status, parental behaviors, and adolescent behaviors were associated with adult chronic inflammation via health characteristics and socioeconomic status in adulthood which subsequently raised mortality risk. Gender moderated the mediating influence of childhood socioeconomic status via unhealthy behaviors and parental behaviors via adult socioeconomic status. Notably, females generally had higher levels of inflammation but lower mortality risk.
**Slavich & Sacher, 2019 [66]**	This review found depression is strongly predicted by early life stress and comorbid with anxiety disorders and certain physical disease conditions, including chronic pain. Excesses in maternal glucocorticoids and abnormalities in immunologic activity have been found to have sex-dependent effects on foetal brain circuits that regulate mood, autonomic activity, blood pressure, and metabolism, resulting in recurrent major depressive disorder across the lifespan characterised by autonomic dysfunction, dysregulated immunologic stress reactivity, and cardiometabolic dysregulation later in life. Research suggests that ovarian hormone fluctuations modulate female susceptibility to stress, brain structure and function, and inflammatory activity and reactivity.
**Samplin et al., 2013 [67]**	The study investigated the association of childhood maltreatment to hippocampal volumes in 67 Caucasian healthy adults (30 males, 37 females, 36.94±14.77 yrs) were assessed for a history of childhood emotional abuse, emotional neglect and physical abuse and received high resolution structural MR imaging scans. Total hippocampal volume, general cognitive ability and subclinical psychopathology were measure and compared. A significant correlational exists between overall childhood trauma score and hippocampus volumes. A positive history of emotional abuse was significantly associated with total hippocampal volume in males but not in females. When the left and right hippocampus were separately assessed, the interaction between emotional abuse and sex was only significant for the left hippocampus. The study suggests that while females may be more resilient to the neurological effects of childhood maltreatment, they are not more resilient to the psychiatric symptoms associated with childhood maltreatment.
**Sandman et al., 2013 [34]**	A longitudinal prospective study evaluated the evidence for sex differences in foetal programming, assessed by summarising previously published sex difference findings (6 studies) and new analyses of previously published findings in which sex differences were not reported (6 studies). Maternal cortisol was evaluated in 125 mother-infant pairs, and infant mental and motor development was assessed at 12 months of age using the Bayley Scales of Infant Development. Data revealed that negative association between prenatal maternal cortisol and 12 month mental development was significant only among male infants, suggesting that males are more susceptible to the effects of early maternal cortisol on developmental delays during infancy. However, females also are influenced by exposure to early adversity. Female foetal exposure to psychobiological stress selectively influences fear/anxiety which persists into preadolescence. Male exposure to early adversity is associated with increased mortality, effectively culling the weak and creating a surviving cohort of the fittest. Females adjust to early adversity but with increased risk for anxiety and affective problems.
**Shalev et al., 2014 [75]**	A longitudinal study that tested the association between the persistence of internalizing disorders and leukocyte telomere length (LTL) in the prospective-longitudinal Dunedin Study (N=1037). Analyses showed that the persistence of internalizing disorders across repeated assessments from ages 11 to 38 years predicted shorter LTL at age 38 years in a dose-response manner, specifically in males. Additional analyses using DNA from blood collected at ages 26 and 38 years showed that LTL erosion was accelerated among males who were diagnosed with internalizing disorder in the interim. No significant associations were found among females in any analysis, highlighting potential sex differences in internalizing-related telomere biology.
**Ujhelyi et al., 2021 [81]**	Carried out a representative survey research in Hungary and in Central–Eastern Europe to assess the prevalence of adverse childhood experiences (ACEs) among adults. 1200 persons aged from 18 years to 112 years (37.65% of the respondents were male). 25% (n = 293) of adults reported any childhood adversity; 5% (n = 59) of them had four or more ACEs. There were no significant gender differences regarding the co-occurrence of ACEs, however among females, emotional abuse and physical abuse were more prevalent (7% (n = 51) for emotional abuse and 6% (n = 44) for physical abuse) than among males (4% (n = 18) for physical abuse and 3% (n = 13) for emotional abuse).

## Data Availability

All data are included in the publication.

## References

[1] Henao-Pérez M., López-Medina D.C., Arboleda A., Bedoya Monsalve S., Zea J.A. (2022). Patients with Fibromyalgia, Depression, and/or Anxiety and Sex Differences. Am. J. Men’s Health.

[2] Söreskog E., Jacobson T., Kirketeig T., Fritzell P., Karlsten R., Zethraeus N., Borgström F. (2023). Impact of spinal cord stimulation on sick leave and disability pension in patients with chronic neuropathic pain: A real-world evidence study in Sweden. Pain.

[3] Arout C.A., Sofuoglu M., Bastian L.A., Rosenheck R.A. (2018). Gender Differences in the Prevalence of Fibromyalgia and in Concomitant Medical and Psychiatric Disorders: A National Veterans Health Administration Study. J. Women’s Health.

[4] Cabañero D., Villalba-Riquelme E., Fernández-Ballester G., Fernández-Carvajal A., Ferrer-Montiel A. (2022). ThermoTRP channels in pain sexual dimorphism: New insights for drug intervention. Pharmacol. Ther..

[5] Chandan J.S., Keerthy D., Zemedikun D.T., Okoth K., Gokhale K.M., Raza K., Bandyopadhyay S., Taylor J., Nirantharakumar K. (2020). The association between exposure to childhood maltreatment and the subsequent development of functional somatic and visceral pain syndromes. EClinicalMedicine.

[6] de Kruijf M., Bos D., Huygen F.J., Niessen W.J., Tiemeier H., Hofman A., Uitterlinden A.G., Vernooij M.W., Ikram M.A., van Meurs J.B. (2016). Structural Brain Alterations in Community Dwelling Individuals with Chronic Joint Pain. AJNR Am. J. Neuroradiol..

[7] Mejía-Terrazas G.E., López-Muñoz E., Hidalgo-Bravo A., Santamaria-Olmedo M.G., Valdés-Flores M. (2022). Association between CACNG2 polymorphisms (rs4820242, rs2284015 and rs2284017) and chronic peripheral neuropathic pain risk in a Mexican population. Eur. Rev. Med. Pharmacol. Sci..

[8] Melikoglu M.A., Celik A. (2017). Does Neuropathic Pain Affect the Quality of Sleep?. Eurasian J. Med..

[9] Vagaska E., Litavcova A., Srotova I., Vlckova E., Kerkovsky M., Jarkovsky J., Bednarik J., Adamova B. (2019). Do lumbar magnetic resonance imaging changes predict neuropathic pain in patients with chronic non-specific low back pain?. Medicine.

[10] Negrón-Blanco L., de Pedro-Cuesta J., Almazán J., Rodríguez-Blázquez C., Franco E., Damián J., DISCAP-ARAGON Research Group (2016). Prevalence of and factors associated with homebound status among adults in urban and rural Spanish populations. BMC Public Health.

[11] Di Pietro F., Lee B., Henderson L.A. (2020). Altered resting activity patterns and connectivity in individuals with complex regional pain syndrome. Hum. Brain Mapp..

[12] Kodila Z.N., Shultz S.R., Yamakawa G.R., Mychasiuk R. (2023). Critical Windows: Exploring the Association Between Perinatal Trauma, Epigenetics, and Chronic Pain. Neurosci. Rev. J. Bringing Neurobiol. Neurol. Psychiatry.

[13] Toriyama T., Horiuchi T., Hongo K. (2017). Characterization of migraineurs presenting interictal widespread pressure hyperalgesia identified using a tender point count: A cross-sectional study. J. Headache Pain.

[14] Häuser W., Hoffmann E.M., Wolfe F., Worthing A.B., Stahl N., Rothenberg R., Walitt B. (2015). Self-reported childhood maltreatment, lifelong traumatic events and mental disorders in fibromyalgia syndrome: A comparison of US and German outpatients. Clin. Exp. Rheumatol..

[15] Jiang L., D’Souza R.S., Oh T., Vincent A., Mohabbat A.B., Ashmore Z., Mauck W.D., Ge L., Whipple M.O., McAllister S.J. (2020). Sex-Related Differences in Symptoms and Psychosocial Outcomes in Patients with Fibromyalgia: A Prospective Questionnaire Study. Mayo Clinic proceedings. Innov. Qual. Outcomes.

[16] Neville S.J., Clauw A.D., Moser S.E., Urquhart A.G., Clauw D.J., Brummett C.M., Harte S.E. (2018). Association Between the 2011 Fibromyalgia Survey Criteria and Multisite Pain Sensitivity in Knee Osteoarthritis. Clin. J. Pain.

[17] Ruschak I., Montesó-Curto P., Rosselló L., Aguilar Martín C., Sánchez-Montesó L., Toussaint L. (2023). Fibromyalgia Syndrome Pain in Men and Women: A Scoping Review. Healthcare.

[18] Staud R., Boissoneault J., Lai S., Mejia M.S., Ramanlal R., Godfrey M.M., Stroman P.W. (2021). Spinal cord neural activity of patients with fibromyalgia and healthy controls during temporal summation of pain: An fMRI study. J. Neurophysiol..

[19] Herringa R.J., Birn R.M., Ruttle P.L., Burghy C.A., Stodola D.E., Davidson R.J., Essex M.J. (2013). Childhood maltreatment is associated with altered fear circuitry and increased internalizing symptoms by late adolescence. Proc. Natl. Acad. Sci. USA.

[20] Banihashemi L., Peng C.W., Verstynen T., Wallace M.L., Lamont D.N., Alkhars H.M., Yeh F.C., Beeney J.E., Aizenstein H.J., Germain A. (2021). Opposing relationships of childhood threat and deprivation with stria terminalis white matter. Hum. Brain Mapp..

[21] Segura-Jiménez V., Estévez-López F., Soriano-Maldonado A., Álvarez-Gallardo I.C., Delgado-Fernández M., Ruiz J.R., Aparicio V.A. (2016). Gender Differences in Symptoms, Health-Related Quality of Life, Sleep Quality, Mental Health, Cognitive Performance, Pain-Cognition, and Positive Health in Spanish Fibromyalgia Individuals: The Al-Ándalus Project. Pain Res. Manag..

[22] Watanabe K., Watanabe M., Takao C., Hong C., Liu Z., Suga T., Tu T.T.H., Sakamoto J., Umezaki Y., Yoshikawa T. (2021). Clinical Characteristics of Predominantly Unilateral Oral Cenesthopathy with and Without Neurovascular Contact. Front. Neurol..

[23] Yeung E.W., Davis M.C., Ciaramitaro M.C. (2016). Cortisol Profile Mediates the Relation Between Childhood Neglect and Pain and Emotional Symptoms among Patients with Fibromyalgia. Ann. Behav. Med. Publ. Soc. Behav. Med..

[24] Zhang H., Lian Y., Xie N., Cheng X., Chen C., Xu H., Zheng Y. (2019). Factors affecting the therapeutic effect of botulinum toxin A on trigeminal neuralgia: A follow-up retrospective study of 152 patients. Exp. Ther. Med..

[25] Bath K.G. (2020). Synthesizing Views to Undestand Sex Differences in Response to Early Life Adversity. Trends Neurosci..

[26] Blasi V., Pirastru A., Cabinio M., Di Tella S., Laganà M.M., Giangiacomo A., Baglio G., Zanette M., Canevini M.P., Walder M. (2020). Early Life Adversities and Borderline Intellectual Functioning Negatively Impact Limbic System Connectivity in Childhood: A Connectomics-Based Study. Front. Psychiatry.

[27] Antoniou G., Lambourg E., Steele J.D., Colvin L.A. (2023). The effect of adverse childhood experiences on chronic pain and major depression in adulthood: A systematic review and meta-analysis. Br. J. Anaesth..

[28] Tan A.C., Jaaniste T., Champion D. (2019). Chronic Widespread Pain and Fibromyalgia Syndrome: Life-Course Risk Markers in Young People. Pain Res. Manag..

[29] Lu X.W., Guo H., Sun J.R., Dong Q.L., Zhao F.T., Liao X.H., Zhang L., Zhang Y., Li W.H., Li Z.X. (2018). A shared effect of paroxetine treatment on gray matter volume in depressive patients with and without childhood maltreatment: A voxel-based morphometry study. CNS Neurosci. Ther..

[30] Meyers J.L., Lowe S.R., Eaton N.R., Krueger R., Grant B.F., Hasin D. (2015). Childhood maltreatment, 9/11 exposure, and latent dimensions of psychopathology: A test of stress sensitization. J. Psychiatr. Res..

[31] Hellou R., Häuser W., Brenner I., Buskila D., Jacob G., Elkayam O., Aloush V., Ablin J.N. (2017). Self-Reported Childhood Maltreatment and Traumatic Events among Israeli Patients Suffering from Fibromyalgia and Rheumatoid Arthritis. Pain Res. Manag..

[32] Osborne N.R., Anastakis D.J., Kim J.A., El-Sayed R., Cheng J.C., Rogachov A., Hemington K.S., Bosma R.L., Fauchon C., Davis K.D. (2021). Sex-Specific Abnormalities and Treatment-Related Plasticity of Subgenual Anterior Cingulate Cortex Functional Connectivity in Chronic Pain. Front. Pain Res..

[33] Achenbach J., Rhein M., Gombert S., Meyer-Bockenkamp F., Buhck M., Eberhardt M., Leffler A., Frieling H., Karst M. (2019). Childhood traumatization is associated with differences in TRPA1 promoter methylation in female patients with multisomatoform disorder with pain as the leading bodily symptom. Clin. Epigenetics.

[34] Sandman C.A., Glynn L.M., Davis E.P. (2013). Is there a viability-vulnerability tradeoff? Sex differences in fetal programming. J. Psychosom. Res..

[35] Ganguly P., Brenhouse H.C. (2015). Broken or maladaptive? Altered trajectories in neuroinflammation and behavior after early life adversity. Dev. Cogn. Neurosci..

[36] Dionisio S., Mayoglou L., Cho S.M., Prime D., Flanigan P.M., Lega B., Mosher J., Leahy R., Gonzalez-Martinez J., Nair D. (2019). Connectivity of the human insula: A cortico-cortical evoked potential (CCEP) study. Cortex.

[37] Echeverri S., Guthrie A.J., Perez D.L. (2022). Toward a possible trauma subtype of functional neurological disorder: Impact on symptom severity and physical health. Front. Psychiatry.

[38] Camilleri M. (2020). Sex as a biological variable in irritable bowel syndrome. Neurogastroenterol. Motil. Off. J. Eur. Gastrointest. Motil. Soc..

[39] Conversano C., Ciacchini R., Orrù G., Bazzichi M.L., Gemignani A., Miniati M. (2021). Gender differences on psychological factors in fibromyalgia: A systematic review on the male experience. Clin. Exp. Rheumatol..

[40] Hruschak V., Flowers K.M., Azizoddin D.R., Jamison R.N., Edwards R.R., Schreiber K.L. (2021). Cross-sectional study of psychosocial and pain-related variables among patients with chronic pain during a time of social distancing imposed by the coronavirus disease 2019 pandemic. Pain.

[41] Nakua E.K., Otupiri E., Dzomeku V.M., Owusu-Dabo E., Agyei-Baffour P., Yawson A.E., Folson G., Hewlett S. (2015). Gender disparities of chronic musculoskeletal disorder burden in the elderly Ghanaian population: Study on global ageing and adult health (SAGE WAVE 1). BMC Musculoskelet. Disord..

[42] Petersen M.W., Schröder A., Jørgensen T., Ørnbøl E., Meinertz Dantoft T., Eliasen M., Benros M.E., Fink P. (2020). Irritable bowel, chronic widespread pain, chronic fatigue and related syndromes are prevalent and highly overlapping in the general population: DanFunD. Sci. Rep..

[43] Slapšinskaitė A., Hristovski R., Razon S., Balagué N., Tenenbaum G. (2017). Metastable Pain-Attention Dynamics during Incremental Exhaustive Exercise. Front. Psychol..

[44] Weimer M.B., Macey T.A., Nicolaidis C., Dobscha S.K., Duckart J.P., Morasco B.J. (2013). Sex differences in the medical care of VA patients with chronic non-cancer pain. Pain Med..

[45] Zhang J., Zhao T., Zhang J., Zhang Z., Li H., Cheng B., Pang Y., Wu H., Wang J. (2022). Prediction of childhood maltreatment and subtypes with personalized functional connectome of large-scale brain networks. Hum. Brain Mapp..

[46] Puetz V.B., Parker D., Kohn N., Dahmen B., Verma R., Konrad K. (2017). Altered brain network integrity after childhood maltreatment: A structural connectomic DTI-study. Hum. Brain Mapp..

[47] Gallo E.A.G., De Mola C.L., Wehrmeister F., Gonçalves H., Kieling C., Murray J. (2017). Childhood maltreatment preceding depressive disorder at age 18 years: A prospective Brazilian birth cohort study. J. Affect. Disord..

[48] Davis E.P., Pfaff D. (2014). Sexually dimorphic responses to early adversity: Implications for affective problems and autism spectrum disorder. Psychoneuroendocrinology.

[49] Page M.J., McKenzie J.E., Bossuyt P.M., Boutron I., Hoffmann T.C., Mulrow C.D., Shamseer L., Tetzlaff J.M., Akl E.A., Brennan S.E. (2021). The PRISMA 2020 statement: An updated guideline for reporting systematic reviews. BMJ.

[50] Jedd K., Hunt R.H., Cicchetti D., Hunt E., Cowell R.A., Rogosch F.A., Toth S.L., Thomas K.M. (2015). Long-term consequences of childhood maltreatment: Altered amygdala functional connectivity. Dev. Psychopathol..

[51] Mansour A., Baria A.T., Tetreault P., Vachon-Presseau E., Chang P.C., Huang L., Apkarian A.V., Baliki M.N. (2016). Global disruption of degree rank order: A hallmark of chronic pain. Sci. Rep..

[52] Li L., Di X., Zhang H., Huang G., Zhang L., Liang Z., Zhang Z. (2022). Characterization of whole-brain task-modulated functional connectivity in response to nociceptive pain: A multisensory comparison study. Hum. Brain Mapp..

[53] Liu J., Liu H., Mu J., Xu Q., Chen T., Dun W., Yang J., Tian J., Hu L., Zhang M. (2017). Altered white matter microarchitecture in the cingulum bundle in women with primary dysmenorrhea: A tract-based analysis study. Hum. Brain Mapp..

[54] Zhou Q., Li M., Fan Q., Chen F., Jiang G., Wang T., He Q., Fu S., Yin Y., Lin J. (2022). Cerebral perfusion alterations in patients with trigeminal neuralgia as measured by pseudo-continuous arterial spin labeling. Front. Neurosci..

[55] Ho T.C., Dennis E.L., Thompson P.M., Gotlib I.H. (2018). Network-based approaches to examining stress in the adolescent brain. Neurobiol. Stress.

[56] Čeko M., Frangos E., Gracely J., Richards E., Wang B., Schweinhardt P., Catherine Bushnell M. (2020). Default mode network changes in fibromyalgia patients are largely dependent on current clinical pain. NeuroImage.

[57] Erpelding N., Simons L., Lebel A., Serrano P., Pielech M., Prabhu S., Becerra L., Borsook D. (2016). Rapid treatment-induced brain changes in pediatric CRPS. Brain Struct. Funct..

[58] Sokołowski A., Folkierska-Żukowska M., Jednoróg K., Moodie C.A., Dragan W.Ł. (2020). The relationship between early and recent life stress and emotional expression processing: A functional connectivity study. Cogn. Affect. Behav. Neurosci..

[59] Taylor R.L., Cooper S.R., Jackson J.J., Barch D.M. (2020). Assessment of Neighborhood Poverty, Cognitive Function, and Prefrontal and Hippocampal Volumes in Children. JAMA Netw. Open.

[60] Wang L., Dai Z., Peng H., Tan L., Ding Y., He Z., Zhang Y., Xia M., Li Z., Li W. (2014). Overlapping and segregated resting-state functional connectivity in patients with major depressive disorder with and without childhood neglect. Hum. Brain Mapp..

[61] Malfliet A., De Pauw R., Kregel J., Coppieters I., Meeus M., Roussel N., Danneels L., Cagnie B., Nijs J. (2019). Gender Differences in the Association of Brain Gray Matter and Pain-Related Psychosocial Characteristics. Pain Physician.

[62] Larson A.A., Pardo J.V., Pasley J.D. (2014). Review of overlap between thermoregulation and pain modulation in fibromyalgia. Clin. J. Pain.

[63] Hanson J.L., Knodt A.R., Brigidi B.D., Hariri A.R. (2018). Heightened connectivity between the ventral striatum and medial prefrontal cortex as a biomarker for stress-related psychopathology: Understanding interactive effects of early and more recent stress. Psychol. Med..

[64] Erpelding N., Sava S., Simons L.E., Lebel A., Serrano P., Becerra L., Borsook D. (2014). Habenula functional resting-state connectivity in pediatric CRPS. J. Neurophysiol..

[65] Nugent A.C., Farmer C., Evans J.W., Snider S.L., Banerjee D., Zarate C.A. (2019). Multimodal imaging reveals a complex pattern of dysfunction in corticolimbic pathways in major depressive disorder. Hum. Brain Mapp..

[66] Slavich G.M., Sacher J. (2019). Stress, sex hormones, inflammation, and major depressive disorder: Extending Social Signal Transduction Theory of Depression to account for sex differences in mood disorders. Psychopharmacology.

[67] Samplin E., Ikuta T., Malhotra A.K., Szeszko P.R., Derosse P. (2013). Sex differences in resilience to childhood maltreatment: Effects of trauma history on hippocampal volume, general cognition and subclinical psychosis in healthy adults. J. Psychiatr. Res..

[68] Younis S., Hougaard A., Noseda R., Ashina M. (2019). Current understanding of thalamic structure and function in migraine. Cephalalgia Int. J. Headache.

[69] Martinez-Torteya C., Muzik M., McGinnis E.W., Rosenblum K.L., Bocknek E.L., Beeghly M., DeCator D., Abelson J.L. (2015). Longitudinal examination of infant baseline and reactivity cortisol from ages 7 to 16 months. Dev. Psychobiol..

[70] Doménech-García V., Peirotén A.R., Imaz M.L., Palsson T.S., Herrero P., Bellosta-López P. (2022). Not just sensitization: Sympathetic mechanisms contribute to expand experimental referred pain. Korean J. Pain.

[71] Lurie D.I. (2018). An Integrative Approach to Neuroinflammation in Psychiatric disorders and Neuropathic Pain. J. Exp. Neurosci..

[72] McQuaid R.J., Gabrys R.L., McInnis O.A., Anisman H., Matheson K. (2019). Understanding the Relation Between Early-Life Adversity and Depression Symptoms: The Moderating Role of Sex and an Interleukin-1β Gene Variant. Front. Psychiatry.

[73] Fischer S., Markert C., Strahler J., Doerr J.M., Skoluda N., Kappert M., Nater U.M. (2018). Thyroid Functioning and Fatigue in Women with Functional Somatic Syn–romes—Role of Early Life Adversity. Front. Physiol..

[74] Enokido M., Suzuki A., Sadahiro R., Matsumoto Y., Kuwahata F., Takahashi N., Goto K., Otani K. (2014). Parental care influences leukocyte telomere length with gender specificity in parents and offsprings. BMC Psychiatry.

[75] Shalev I., Moffitt T.E., Braithwaite A.W., Danese A., Fleming N.I., Goldman-Mellor S., Harrington H.L., Houts R.M., Israel S., Poulton R. (2014). Internalizing disorders and leukocyte telomere erosion: A prospective study of depression, generalized anxiety disorder and post-traumatic stress disorder. Mol. Psychiatry.

[76] Ehrlich K.B., Miller G.E., Rogosch F.A., Cicchetti D. (2021). Maltreatment exposure across childhood and low-grade inflammation: Considerations of exposure type, timing, and sex differences. Dev. Psychobiol..

[77] Morton P.M., Ferraro K.F. (2020). Early Social Origins of Biological Risks for Men and Women in Later Life. J. Health Soc. Behav..

[78] Smith M.T., Remeniuk B., Finan P.H., Speed T.J., Tompkins D.A., Robinson M., Gonzalez K., Bjurstrom M.F., Irwin M.R. (2019). Sex differences in measures of central sensitization and pain sensitivity to experimental sleep disruption: Implications for sex differences in chronic pain. Sleep.

[79] Merrick M.T., Ford D.C., Ports K.A., Guinn A.S. (2018). Prevalence of Adverse Childhood Experiences From the 2011-2014 Behavioral Risk Factor Surveillance System in 23 States. JAMA Pediatr..

[80] Radcliff E., Crouch E., Strompolis M. (2018). Rural-urban differences in exposure to adverse childhood experiences among South Carolina adults. Rural Remote Health.

[81] Ujhelyi Nagy A., Kuritár Szabó I., Hann E., Kósa K. (2019). Measuring the Prevalence of Adverse Childhood Experiences by Survey Research Methods. Int. J. Environ. Res. Public Health.

